# Supplementary material for: WordCommentsAnalyzer: A windows software tool for qualitative research
Source: F1000Res. 2018 Sep 4;7:536. Originally published 2018 May 3. [Version 2] doi: 10.12688/f1000research.14819.2 (PMC6137411; doi:10.12688/f1000research.14819.2)
Supplement: Supplementary file 3 [file f1000research-7-17614-s0002.tgz › b2a79496-4ada-47c4-9682-a99ce867836d.zip!/Eight Iranian Health Journals/Salmand_Iranian Journal of Ageing _45.docx]

© Salmand_Iranian Journal of Ageing . This work is licensed under CC BY-NC 4.0 (https://creativecommons.org/licenses/by-nc/4.0/).

Volume & Issue: دوره 2 - شماره 1

Title: بیماری آلزایمر: دیروز، امروز، فردا

http://salmandj.uswr.ac.ir/article-1-9-fa.html

بیماری آلزایمر شایع‌ترین و شناخته‌شده‌ترین علت خردزدودگی (دمانس) است، یک نابسامانی پیش‌رونده و برگشت‌ناپذیر مغزی که بر کارکرد شناختی شخصیت، اندیشه، ادراک و رفتار تأثیر می‌گذارد. بیماری آلزهایمر چهارمین علت مرگ در کشورهای غربی است. جالب است بدانیم که این بیماری تا یکصد سال پیش از این برای جامعه پزشکی ناشناخته بود و مشخصاً نامی هم نداشت. دکتر آلوییز آلزهایمر با بررسی‌های دقیق بالینی روانپزشکی، نوروپاتولوژیک و نوروآناتومیک چند مورد، ازجمله نخستین مورد شناخته‌شده این بیماری که خانمی موسوم به آگوسته دتر بود، وجود این بیماری را حدس زد و مستند نمود. سپس امیل کرپلین با آگاهی از مواردی که دکتر آلزایمر بررسی کرده بود و نیز موارد دیگری که در نخستین دهه قرن بیستم از این بیماری منتشر شده بود، نام آلزایمر را بر این بیماری نهاد. توصیف‌های آلزایمر و کرپلین با آنچه امروز به عنوان بیماری آلزایمر می‌دانیم مطابقت دارد. میکروسکوپ الکترونی، شکل‌شناسی کمّی و بیوشیمی پیشرفته از نیمه دوم قرن بیستم میلادی تاکنون دوره جدیدی را در پژوهش‌های مربوط به دمانس با توصیف فوق ساختاری و بیوشیمیایی از پلاک‌های پیری و نوروفیبریلری که نشانگرهای عمده بیماری آلزایمر هستند، گشوده است. پژوهش‌های بنیادی موجب بینش به ژنتیک مولکولی و پاتوفیزیولوژی بیماری آلزایمر شد و بر پایه یافته‌های بیوشیمیایی گزینش‌های نوین درمان داروشناختی ممکن شد. کوشش‌های آینده احتمالاً بیشتر بر پیشگیری از وقوع بیماری متمرکز است. استرس اکسیداتیو، یون‌های فلزی و پروتئین‌های تجمع‌یافته نابجا و التهاب همه از مواردی است که احتمالاً در سبب‌شناسی آلزایمر نقش دارند و پژوهش‌های آینده متمرکز بر شناخت بهتر و جلوگیری از روی‌دادن آن‌ها خواهد بود و سرانجام اینکه پیوند سلول‌های بنیادی که در جانوران منجر به بهبود چشمگیر در کارکرد مغز شده است می‌تواند راهگشای درمان بیماری آلزایمر در آینده باشد.

Title: سیستم خدمات ویژه سالمندی در کشورهای آمریکا و ژاپن و ارایه شاخص هایی جهت تدوین برنامه راهبردی خدمات سالمندی در ایران

http://salmandj.uswr.ac.ir/article-1-1236-fa.html

سالمندی می تواند دوران سرزندگی، نشاط، عزم، اراده، بزرگی، عزت و شکوه باشد. از بارزترین تغییرات جمعیت شناسی قرن بیست و یکم سالخوردگی جمعیت است که تمامی کشورهای جهان منجمله ایران را درگیر کرده است. کشورهای پیشرفته در راستای حل پیامدهای سالخوردگی چاره ای اندیشیده اند اما اگر کشورهای جهان سوم منجمله ایران (حدود 8% جمعیت بالای 60 سال) سیاست های لازم جهت برنامه ریزی خدمات مراقبتی سالمندان بکار نگیرند، در آینده نزدیک با بحران اجتماعی- اقتصادی- بهداشتی و درمانی مواجه خواهند شد. هر برنامه اجرایی سالمندی و سلامت سالمندی باید بر سه پایه استوار باشد: (1 افراد سالمند و توسعه (شرکت دادن) که بر نیاز جامعه متمرکز می شود تا سیاست هایشان را با آن منطبق کرده و موسسات بخاطر منافع جامعه رشد جمعیت سالمندی را با نگاه نیروی فعال ببینند. (2 ارتقای سلامت و رفاه (سلامت همه جانبه) که تاکید بر نیاز سیاست هایی که سلامت با نشاط را از دوران کودکی تا طول زندگی ارتقا داده تا در زمان سالمندی دوران سالمی داشته باشند. (3 تضمین توانایی و حمایت محیطی (امنیت) که سیاست های مبتنی بر خانواده و جامعه را ارتقا داده و بنیان سالمندی ایمن را فراهم می آورد. در این راستا کشور ژاپن طرح طلایی (gold plan) و بیمه مراقبتی درازمدت و طرح فرشته (Angel) را برای کاهش میزان زاد و ولد بکار گرفته و کشور آمریکا با توجه به شاخص هایی برنامه راهبردی خود را تهیه کرده است که در برگیرنده ابعاد مختلف اقتصادی، رفاهی، بهداشتی، و درمانی است. در این مقاله با مروری بر سیستم عملکردی کشورهای پیشرفته (ژاپن و آمریکا) و الهامگیری از نقاط مثبت و با نگاه به تجارب آنها در این امر، شاخص ها و سرفصل هایی را جهت تدوین نظام جامع خدمات ویژه سالمندان پیشنهاد کرده است.

Title: مشکلات اجتماعی خانواده‌های مراقبت‌کننده از سالمند ناتوان در شهر کرج

http://salmandj.uswr.ac.ir/article-1-10-fa.html

اهداف: یکی از چالش های مهم در جوامع امروز افزایش جمعیت سالمند و در نتیجه آن افزایش ناتوانی است. به همین دلیل مراقبت از این قشر نیز یک امر مهم در سیستم های بهداشتی می باشد. بیشتر مسوولیت مراقبت از سالمند بر عهده اعضای خانواده می باشد. این خانواده ها برای برآوردن نیازهای عضو سالمند با مشکلات عدیده ای مواجه می باشند.هدف از این مطالعه بررسی مشکلات اجتماعی خانواده های دارای سالمند ناتوان مراجعه کننده به درمانگاه های بیمارستان های دولتی شهر کرج در سال 1381 بوده است. مواد و روش‌ها: پژوهش حاضر یک مطالعه توصیفی همبستگی است. به همین دلیل تعداد 90 خانواده که به مراکز مورد پژوهش مراجعه کرده بودند به روش نمونه گیری در دسترس و مبتنی بر هدف انتخاب گردیدند. ابزار گردآوری داده ها پرسش‌نامه بود که با حضور پژوهشگر در منازل واحدهای مورد پژوهش و مصاحبه با تمام اعضای خانواده ها تکمیل گردید. یافته‌ها: پس از تجزیه‌وتحلیل داده‌ها نتایج حاصله از این پژوهش نشان داد که میانگین سن سالمندان بیمار 77 سال بود که اکثرا به علت بیماری‌های قلبی- عروقی به بیمارستان مراجعه کرده بودند. همچنین میانگین امتیاز استقلال سالمندان در انجام فعالیت‌های روزانه 46 بود. یعنی سالمندان بیمار در این پژوهش در انجام فعالیت‌های روزانه زندگی، وابستگی نسبی داشتند. به طور کلی زنان روزانه بیشترین کمک جسمانی، عاطفی، اجتماعی را به سالمندان ارائه می‌دهند. اکثر خانواده‌ها، مشکلات در ارتباطات خارجی را در حد متوسط تا زیاد، مشکلات در گذراندن اوقات فراغت را در حد متوسط تا زیاد و مشکلات اقتصادی را نیز در حد متوسط تا زیاد بیان کرده و تنها درصد کمی از آن‌ها دارای مشکلات در حد کم بوده اند. در مورد مشکلات اشتغال به کار، یافته‌ها نشان داد که کلیه زنان مشکلاتشان را در حد زیاد تا متوسط و اکثر مردان مشکلاتشان را در حد متوسط تا کم بیان کرده اند. نتایج پژوهش نشان داد که به‌طور کلی مشکلات اجتماعی زنان بیش از مردان می‌باشد. همچنین با افزایش سن سالمند، افزایش وابستگی سالمند در انجام فعالیت‌های روزانه، افزایش اختلال ذهنی سالمند و افزایش مدت زمان کمک جسمانی با سالمند، مشکلات اجتماعی خانواده‌های دارای سالمند بیمار افزایش خواهد یافت. نتیجه‌گیری: نتایج این مطالعه بیانگر این نکته است که با توجه به افزایش جمعیت سالمند و افزایش امید به زندگی در جامعه ما، باید هر چه سریع‌تر به طراحی و اجرای برنامه‌های حمایتی برای سالمندان و خانواده‌هایشان بپردازیم. در صورت تداوم مراقبت از سالمندان وابسته و عدم حمایت دولت از این خانواده ها، امکان خستگی و افزایش بار مسئولیت در این خانواده ها وجود دارد که این مساله منجر به سوء رفتار با فرد سالمند می‌گردد. علی رغم وجود مشکلات ذکر شده در این خانواده‌ها، می توان با حمایت از آنان به‌خصوص زنان، از افزایش اختلال در روابط اعضای خانواده ها با سالمندان و فعالیت‌های اجتماعی‌شان و فرستادن سالمندان به سرای سالمندان جلوگیری نمود.

Title: کیفیت زندگی در سالمندان عضو کانون‌های فرهنگسرای سالمندان شهر تهران

http://salmandj.uswr.ac.ir/article-1-11-fa.html

اهداف: سالمندان یکی از گروه‌های اجتماعی رو به رشد در جوامع امروزی هستند که با توجه به هرم سنی کشور اهمیت روز افزونی یافته‌اند و هرگونه تصمیم‌گیری و برنامه‌ریزی در رابطه با این گروه نیازمند شناخت دقیق و مبتنی بر مشاهدات عینی و علمی مسائل آنان است. از طرفی در جهان کنونی، یکی از شاخص‌های ارزشمند، برای ارزیابی در زمینه‌های توانبخشی، بهداشتی، درمانی و... کیفیت زندگی است. مواد و روش‌ها: هدف از این تحقیق مقطعی و توصیفی، تحلیلی بررسی کیفیت زندگی سالمندان شهر تهران است. جامعه پژوهش، سالمندان عضو فرهنگسرای سالمند بود که 300 نفر نمونه، به‌صورت تصادفی از افراد 60 سال و بالاتر انتخاب شدند و اهداف پژوهش در قالب شاخص‌های کیفیت زندگی مطرح گردیدند. اطلاعات از طریق پرسش‌نامه استاندارد WHOQOL-BREF با چهار حیطه جسمانی، روانی، اجتماعی و محیطی و با روش مصاحبه رو در رو، جمع‌آوری شد و به منظور تجزیه‌وتحلیل اطلاعات حاصله، از آزمون‌های Anova، T.Test و رگرسیون با استفاده از SPSS انجام گردید. یافته‌ها: بر اساس یافته ها، 223 نفر زن (74/33درصد) و 77 نفر مرد (25/66درصد) با دامنه سنی 60 تا 85 و میانگین سنی 68/08 سال در مطالعه شرکت داشتند. نتایج پژوهش حاکی از این بود که بین مردها و زن‌ها، کیفیت زندگی فقط در حیطه جسمانی (0/001>P) و روانی (0/014>P) تفاوت معنی‌دار و امتیاز در مردها بالاتر از زن‌ها بود. در بررسی رابطه بین کیفیت زندگی و سن نیز، فقط در حیطه جسمانی (0/036>P) و روانی (0/097>P) اختلاف معنی‌دار بوده و در سایر حیطه‌ها اختلاف معنی‌داری مشاهده نشد. در ارتباط کیفیت زندگی و وضعیت تاهل، افراد متاهل نسبت به کسانی که همسر خود را از دست داده‌اند، کیفیت زندگی بهتری داشتند و افرادی که هرگز ازدواج نکرده‌اند از سلامت جسمانی بالاتری برخوردار بودند. و هرقدر بر میزان تحصیلات افزوده شده، کیفیت زندگی نیز افزایش یافته و ابتلا به بیماری نیز با کیفیت زندگی در همه حیطه‌ها تفاوت معنی‌دار را نشان داد. نتیجه‌گیری: یافته‌های فوق نشان داد که فاکتورها و عوامل مختلفی مانند سن، جنس، وضعیت تاهل، میزان تحصیلات و ابتلا به بیماری‌ها بر کیفیت زندگی سالمندان موثر است و با شناخت این عوامل و انجام اقدامات پیشگیرانه و همزمان و برنامه‌ریزی برای آینده می‌توان بر مشکلات سالمندان فعلی فایق آمده و از مشکلات سالمندان آینده نیز پیشگیری نمود.

Title: نیازهای اوقات فراغت سالمندان شهرستان قزوین

http://salmandj.uswr.ac.ir/article-1-12-fa.html

اهداف: "اوقات" در لغت به معنای زمان ها و "فراغت" به مفهوم آسایش، استراحت، آسودگی و رهایی از کاری است و در اصطلاح عبارت است: فرصت و زمانی که پس از به انجام رساندن کار و شغل روزانه باقی می ماند. جامعه شناسان معتقدند که فراغت واقعیتی است کاملا تازه که با جامعه صنعتی و پدیده شهرنشینی به وجود آمده است و آن را نمی‌توان با بیکاری قرون گذشته مقایسه کرد. این مطالعه برای بررسی نیازهای اوقات فراغت سالمندان انجام شده است. مواد و روش‌ها: بدین منظور پرسش‌نامه‌های به صورت تصادفی بین سالمندان پارک های عمومی شهرستان قزوین توزیع گردید و اطلاعات جامع با استفاده از آمار استنباطی غیر پارامتریک مورد ارزیابی قرار گرفت. علاوه بر آن از آزمون ضریب همبستگی نیز استفاده شد. یافته‌ها: بررسی‌ها نشان می‌دهد که مجموعه برنامه‌های فراغتی ذیل در واقع یک مجموعه نسبتا کامل، هدف‌گرا و اغلب از نوع برنامه‌های فعال در حوزه اوقات فراغت سالمندان است که چنانچه با طراحی و برنامه‌ریزی دقیق و همه جانبه نسبت به اجرا و توسعه آن در سطح کشور اقدام شود نتایج مطلوبی به دنبال خواهد داشت. نتیجه‌گیری: با عنایت به نتایج حاصل از پژوهش از میان برنامه‌های فراغتی سالمندان نمایش فیلم، برنامه‌های ورزشی و هنری بیشتری سهم را به خود اختصاص داده‌اند. نگرش سالمندان نسبت به ورزش بسیار مثبت ارزیابی شد چنانچه پرداختن به ورزشی را در سلامتی جسمی و روحی، فرهنگی، اجتماعی به نحو قابل توجهی موثر دانسته‌اند. همچنین تجهیز کلیه مراکز نگهداری سالمندان به تلویزیون، رادیو و امکانات مناسب جهت نمایش‌دادن فیلم‌های ویدیویی ورزشی و غیر ورزشی و کامل نمودن تاسیسات ورزشی و استفاده از نیروی انسانی متخصص در واحدهای فوق برنامه در بهینه کردن اهداف اوقات فراغت سالمندان بسیار مثمر ثمر خواهد بود.

Title: تاثیر ورزش بر کیفیت زندگی زنان سالمند عضو کانون روزانه جهاندیدگان شهر شیراز (سال 1386)

http://salmandj.uswr.ac.ir/article-1-13-fa.html

اهداف: ناتوانی‌ها و ازکارافتادگی شایع در دوران سالمندی ناشی از مجموعه‌ای از عوامل مختلف است که از مهم‌ترین عوامل آن عدم فعالیت و کم‌تحرکی و عدم استفاده صحیح از عضلات است. کاربرد صحیح برنامه فعالیت می تواند پیشرفت قابل توجهی در کیفیت زندگی سالمندان ایجاد کند. در این مطالعه تاثیر ورزش بر کیفیت زندگی زنان سالمند کانون روزانه جهاندیدگان شهر شیراز در سال 1386 مورد بررسی قرار می گیرد. موارد و روش‌ها: این مطالعه به روش نیمه تجربی در کانون جهاندیدگان شهر شیراز در سال 1386 انجام شده است. جامعه مورد پژوهش در این تحقیق 400 زن سالمند 60-79 سال بود که از این تعداد 232 نفر به روش بلوک های تبدیل شده تصادفی به دو گروه مداخله (116 نفر) و کنترل (116 نفر) تخصیص شدند. گروه مداخله در برنامه ورزشی متناسب سالمندان در مدت 8 هفته (هفته ای سه جلسه و هر جلسه به مدت 30 دقیقه) شرکت کردند. 116 نفر دیگر در گروه کنترل قرار گرفتند. داده های جمع آوری شده در دو مرحله قبل و بعد از برنامه ورزشی در دو گروه مداخله و کنترل به کمک نرم افزار SPSS مورد تجزیه و تحلیل قرار گرفت. در این رابطه از آزمون های آماری کای اسکوئر، من ویتنی، ویلکاکسون، تی زوجی، تی مستقل و تحلیل کوواریانس استفاده شد. در این تحقیق برای سنجش میزان کیفیت زندگی از پرسشنامه اصلاح شده کیفیت زندگی سالمندان لیپاد استفاده شد. یافته‌ها: میانگین نمره کیفیت زندگی قبل از بکارگیری برنامه ورزشی در گروه مداخله 70/60 و در گروه کنترل 70/99 بود. پس از اجرای برنامه ورزشی میانگین نمره کیفیت زندگی در گروه مداخله 75/80 و در گروه کنترل 70/64 بود. نتایج در سطح 95درصد از لحاظ آماری معنی‌دار بود (P=0/013). نتیجه‌گیری: براساس یافته های پژوهش می‌توان نتیجه گرفت انجام ورزش در زنان سالمند گروه مداخله موجب ارتقای سطح کیفیت زندگی ایشان گشته است لذا ورزش موجب بهبودی زندگی زنان سالمند می‌گردد.

Title: تاثیر سمعک بر افسردگی در سالمندان دچار افت شنوایی در مراکز سالمندان شهر تهران

http://salmandj.uswr.ac.ir/article-1-14-fa.html

اهداف: آسیب شنوایی متوسط و شدیدتر در دوره سالمندی می تواند بر سلامت جسمی و روانی- اجتماعی افراد تاثیر بگذارد. این افراد ممکن است دچار ضعف خودباوری، زودرنجی، انزوا طلبی، ناامیدی، افسردگی و احتمالا اختلال فراموشی شوند. مطالعه حاضر با هدف بررسی تاثیر سمعک بر افسردگی سالمندان دچار افت شنوایی در مراکز سالمندان شهر تهران صورت گرفته است. مواد و روش‌ها: در این مطالعه مقطعی، افسردگی در 3 گروه از سالمندان شامل 30 سالمند با شنوایی هنجار، 30 سالمند با افت شنوایی بیش از حد متوسط و بدون سمعک و 20 سالمند با افت شنوایی بیش از حد متوسط و با سمعک مورد بررسی قرار گرفت. برای سنجش افسردگی از پرسش نامه افسردگی بک (BDI-II) استفاده شد و داده ها از طریق آنالیز واریانس یکراهه (ANOVA) مورد تجزیه و تحلیل آماری قرار گرفت. یافته‌ها: میانگین نمرات افسردگی برای 3 گروه به ترتیب 11/43، 21/53 و 16/40 بود که مؤید اختلاف معنی دار بین 3 گروه بود. همبستگی بین سن و افسردگی در گروه با کم شنوایی و بدون سمعک دیده نشد، اما در 2 گروه دیگر این همبستگی مشهود بود. نتیجه‌گیری: به نظر می‌رسد استفاده از سمعک در کاهش افسردگی سالمندان کم‌شنوا موثر باشد.

Title: مقایسه وضعیت سلامت تغذیه‌ای مردان سالمند فعال و غیرفعال شهر شیراز

http://salmandj.uswr.ac.ir/article-1-15-fa.html

اهداف: امروزه با توسعه علوم مختلف، میزان مرگ و میر کاهش یافته و امید به زندگی رو به افزایش است و جمعیت جهان به سمت سالمندی پیش می‌رود. از آنجایی که در این سنین، تغییرات فیزیولوژیکی ایجاد شده روی نیازهای تغذیه ای تاثیر می‌گذارد و با توجه به این که مراقبت‌های تغذیه‌ای این دوران بیشترین نقش را در حفظ و ارتقای سلامت آنان دارد. لذا هدف از این پژوهش بررسی وضعیت سلامت تغذیه ای مردان سالمند فعال و غیرفعال شهر شیراز است. مواد و روش‌ها: این پژوهش به‌صورت توصیفی صورت پذیرفت که بدین منظور تعداد 156 مرد سالمند بالای 60 سال از چهار پارک بزرگ شیراز به‌صورت تصادفی به عنوان نمونه آماری انتخاب شدند که با توجه به اینکه فعالیت بدنی دارند یا خیر، در دو گروه سالمند دارای فعالیت بدنی و سالمند بدون تحرک قرار گرفتند. ابزار مورد استفاده در این پژوهش، پرسش‌نامه بررسی وضعیت سلات است. یافته‌ها: یافته‌ها نشان داد که در مجموع از 156 نفر سالمند مورد مطالعه، 34.61درصد (35 نفر فعال و 19 نفر غیرفعال) از آن‌ها از وضعیت تغذیه‌ای مناسبی برخوردارند و 37/81درصد (28 نفر فعال و 31 نفر غیرفعال) در معرض خطر متوسط برای ابتلا به مشکلات تغذیه‌ای و 27/56درصد (15 نفر فعال و 28 نفر غیرفعال) در معرض خطر بالایی برای ابتلا به مشکلات تغذیه‌ای هستند. نتیجه‌گیری: نتایج حاصل از پژوهش نشان می‌دهد که در مجموع سالمندان از وضعیت تغذیه‌ای مناسبی برخوردار نیستند هرچند که سالمندان فعال از لحاظ وضعیت تغذیه‌ای، در سطح مناسب‌تری نسبت به سالمندان غیرفعال بودند. که این امر خود نشان‌دهنده این است که فعالیت بدنی می‌تواند در تامین سلامت تغذیه‌ای سامندان نقش مثبتی ایفا کند. لذا ضرورت طراحی استراتژی‌های مناسب جهت حمایت و آموزش تغذیه سالمندان با هدف بهبود و اصلاح رژیم آن‌ها، هم چنین ترویج فعالیت بدنی با کمک ارگان‌ها و سازمان‌های مختلف ازجمله رسانه‌های گروهی، پیشنهاد می‌شود.

Title: مشکلات دارودرمانی در سالمندان

http://salmandj.uswr.ac.ir/article-1-16-fa.html

مطالعات نشان داده است که افراد مسن نسبت به دیگر گروه‌های سنی، بیشتر به بیماری‌های مزمن مبتلا شده و تحت درمان هستند. دارو درمانی درافراد مسن رایج است. به طور متوسط هر فرد مسن از 4/5 داروی تجویزی و 2/1 داروی غیرتجویزی استفاده می‌کند و در هر سال تقریبا 12 تا 17 تجویز دارد. البته بیشترین مصرف دارو در بیمارستان ها و مراکز درمانی مراقبتی است. شایع‌ترین داروهای مصرفی، مسکن‌های درد، ضدالتهاب‌های مفصلی، ضدفشار خون، داروهای قلبی- عروقی و آرام‌بخش‌ها هستند. روند طبیعی سالمندی، خود به تنهایی بر لزوم تغییر در شیوه دارو درمانی در افراد سالمند را تاکید می‌کند. به دلیل بروز بیماری‌های مختلف ناشی از افزایش سن، الگوی مصرف دارو و تجویز داروهای متعدد، به‌خصوص در بیمارستان‌ها و خانه‌های سالمندان تغییر می‌کند. گوناگونی و تحولات معمول در شیوه زندگی افراد کهنسال نیز تاثیر عمده‌ای بر روش مصرف دارو در آنان دارد. این مقاله ضمن مروری اجمالی مشکلات درمانی، عوارض جانبی ناخواسته دارویی، تغییرات فیزیولوژیک و اثر کلی این تغییرات بر فارماکوکینتیک و فارماکودینامیک دارویی، پیشنهاداتی را نیز به منظور حفظ سلامتی و بالابردن کیفیت زندگی سالمندان توصیه می‌نماید.
